# Dental Notes

**Published:** 1902-12

**Authors:** William Rollins


### DENTAL NOTES.

BY WILLIAM ROLLINS.

### NOTE VI. ON A FORM OF OXYPHOSPHATE FILLING.

These notes have been interrupted by more important matters. This one is written at the request of Dr. Louis Jack.

The ordinary oxyphosphate of zinc filling is so temporary that any change in its composition which will make it more durable is important. I have found a number of cements which have more lasting properties. One of these has now been tested for nearly twenty-five years. To make it, add oxide of iron in the form of the finest rouge to the oxide of zinc in the proportion of one part of the former to six to ten of the latter. The color is dark red, but this is no objection to its use in many places. The mixture may be quickly produced by shaking the two powders together in a test-tube.

